# Temporal variation in initial UICC stage and post-diagnostic treatment intervals in patients with oral squamous cell carcinoma in Saxony before and during the COVID-19 pandemic

**DOI:** 10.3389/fonc.2026.1770029

**Published:** 2026-10-19

**Authors:** Gerald Stach, Günter Lauer, Samaneh Farahzadi, Tom Alexander Schröder, Michaela Buckova

**Affiliations:** Department of Oral and Maxillofacial Surgery, Faculty of Medicine and University Hospital Carl Gustav Carus, TUD Dresden University of Technology, Dresden, Germany

**Keywords:** COVID-19 pandemic, medical care, oral squamous cell carcinoma, Saxony, TNM staging, UICC classification

## Abstract

**Background:**

Oral squamous cell carcinoma is a malignant tumor for which early diagnosis and timely treatment are important determinants of prognosis. The COVID-19 pandemic disrupted healthcare delivery and reduced participation in preventive examinations. This study examined temporal differences in initial tumor stage and post-diagnostic treatment intervals among patients with oral squamous cell carcinoma.

**Methods:**

This retrospective registry-based study included 3,216 patients diagnosed with oral squamous cell carcinoma between 1 January 2018 and 5 August 2024. Patients were categorized into the pre-pandemic period, the early pandemic period, and the later pandemic period according to their date of diagnosis. Initial UICC stage and post-diagnostic treatment intervals were compared between the three predefined periods and additionally analyzed according to year of diagnosis. Descriptive statistics and non-parametric statistical tests were applied.

**Results:**

The distribution of initial UICC stages differed between the three predefined periods, with a higher proportion of stage IV disease in the pre-pandemic period; however, the association was weak. Significant differences between the predefined periods were also identified for the interval from diagnosis to the first multidisciplinary tumor board and for the intervals from the tumor board to surgery with and without subsequent radiotherapy. In the year-by-year analysis, differences were observed in initial UICC stage and in the intervals from diagnosis to the tumor board and from the tumor board to surgery with subsequent radiotherapy. No significant year-by-year difference was observed for the interval from the tumor board to surgery without subsequent radiotherapy. Overall, the annual patterns were non-linear.

**Conclusion:**

No prolongation of the evaluated post-diagnostic treatment intervals was observed during the early pandemic period compared with the pre-pandemic period. However, the differences in initial UICC stage and selected treatment intervals were small, and the additional year-by-year analysis demonstrated non-linear temporal variation. The findings should therefore be interpreted as temporal associations rather than as evidence of a causal adverse or beneficial effect of the COVID-19 pandemic.

## Introduction

Oral squamous cell carcinoma (OSCC) is the most common malignant neoplasm of the oral cavity and arises from the epithelial cells of the oral mucosa. In Germany, cancers of the oral cavity and pharynx account for a substantial number of newly diagnosed malignancies each year, with men affected more frequently than women (1). Globally, considerable geographic and sex-related variation in the incidence of oral and oropharyngeal cancer has been reported (2). OSCC may arise at different sites of the oral mucosa, including the floor of the mouth, tongue, retromolar region, and other oral subsites (3). Disease progression may involve regional lymph-node metastasis and, in advanced disease, distant metastatic spread (4).

Timely diagnosis and treatment are important components of oral cancer care, although the reported associations between diagnostic or treatment delays, tumor stage, and survival vary between studies (5, 6). Delays may occur at different stages of the diagnostic and treatment pathway and can arise both before and after the first contact with the healthcare system. Earlier studies demonstrated substantial patient-related and healthcare-related delays in patients with oral and head and neck cancer (7–10). Jones et al. reported waiting periods at several stages between initial presentation, histological diagnosis, and definitive treatment, whereas Hollows et al. identified a substantial contribution of patient-related delay before the first medical consultation (9, 10). A systematic review subsequently demonstrated considerable heterogeneity in reported diagnostic and treatment intervals and in their associations with disease stage and survival in oral cavity cancer (6).

The emergence of coronavirus disease 2019 (COVID-19) placed additional pressure on established healthcare pathways. Following the first documented COVID-19 cases in Germany in January 2020 (11), the World Health Organization characterized COVID-19 as a pandemic on 11 March 2020 (12). In Germany and Saxony, measures were subsequently introduced to reduce SARS-CoV-2 transmission and preserve healthcare capacity, including restrictions affecting routine healthcare delivery and the postponement of elective procedures (13).

Changes during the pandemic affected both the provision and utilization of healthcare services. Survey data indicated that patients postponed preventive examinations, cancer screening, and dental visits during the early pandemic period (14). German healthcare data also demonstrated substantial reductions in the utilization of several screening and outpatient services during the first phase of the pandemic (15). In Saxony, clinical cancer registry data showed a temporary decrease in the number of newly documented cancer cases during the early pandemic period, suggesting that pandemic-related changes may also have affected cancer detection and registration (16).

Studies specifically investigating oral and head and neck cancer care during the COVID-19 pandemic have reported heterogeneous findings. In a German cohort of patients with oral cancer, Metzger et al. observed a longer treatment delay and a higher pathological T classification during the first pandemic year compared with the preceding period (17). In contrast, a German multicenter study found no clear increase in tumor stage and reported a trend toward a shorter time to intervention during the lockdown period (18). More recent evidence similarly indicates that the impact of the pandemic was not uniform across healthcare systems. A meta-analysis of head and neck cancer studies suggested a modest shift toward more advanced disease at diagnosis during the pandemic, although considerable heterogeneity between individual studies was observed (19). A systematic review of time to treatment in head and neck cancer likewise demonstrated heterogeneous findings, with individual studies reporting prolonged, shortened, or unchanged treatment intervals during the pandemic (20).

For the present study, the observation period was divided into three predefined pandemic-related periods: a pre-pandemic period before March 2020, an early pandemic period from March 2020 through September 2021, and a later pandemic period after September 2021. September 2021 was selected as a pragmatic transition point rather than as the epidemiological end of the COVID-19 pandemic. By this time, the vaccination programme had been widely implemented and healthcare services had gained substantial experience in maintaining routine care under pandemic conditions (21). The second cut-off was therefore intended to distinguish the initial phase of acute pandemic-related disruption from a later phase characterized by increasing adaptation of healthcare delivery to the continued circulation of SARS-CoV-2. This classification does not imply that the pandemic or its potential effects on cancer care had ended after September 2021.

The aim of this registry-based study was to investigate temporal differences in initial UICC stage and post-diagnostic treatment intervals among patients with OSCC in Saxony before and during the COVID-19 pandemic. We compared the three predefined pandemic-related periods and additionally performed an exploratory year-by-year analysis from 2018 to 2024 to assess whether observed differences reflected consistent changes around the predefined cut-off points or broader temporal variation. Given the retrospective observational design, the analyses were intended to identify temporal associations rather than to establish causal effects of the COVID-19 pandemic.

## Materials and methods

### Patient cohort

The Ethics Committee of the Technical University of Dresden approved the project on November 14, 2023 (BO-EK-433102023).

This retrospective study included 3,216 adult patients who were diagnosed with oral squamous cell carcinoma between 1 January 2018 and 5 August 2024 and reported to the Saxony Cancer Registry. Available registry variables included sex, age, initial TNM classification (T0–T4, N0–N3, and M0–M1), treatment course, and treatment modality (surgery, radiotherapy, surgery and radiotherapy, or systemic therapy). Analyses of individual variables and endpoints were performed using patients with the required information available. The data were checked according to the inclusion criteria and for completeness.

Following extraction of the anonymized registry data, patients were divided into three groups according to the date of diagnosis: the pre-pandemic period (before March 2020), the early pandemic period (March 2020–September 2021), and the later pandemic period (after September 2021).

March 2020 was selected as the first cut-off because the World Health Organization characterized COVID-19 as a pandemic on 11 March 2020. September 2021 was selected as a pragmatic transition point rather than as the epidemiological end of the pandemic. By this time, the vaccination programme had been widely implemented, population immunity resulting from vaccination and previous infection had increased substantially, and healthcare services had gained experience in maintaining routine care under pandemic conditions. The second cut-off was therefore intended to distinguish the initial phase of acute pandemic-related disruption from a later phase characterized by increasing adaptation of healthcare delivery to the continuing circulation of SARS-CoV-2. This classification does not imply that the pandemic or its potential effects on cancer care had ended after September 2021.

Initial UICC stage, classified according to the eighth edition of the TNM classification, was obtained directly from the cancer registry. UICC substages A–C were combined into the corresponding main stages, resulting in the categories UICC stages 0–IV used for the statistical analysis.

### Statistical analysis

The statistical analysis was performed using IBM SPSS Statistics 29.

Continuous variables were summarized using the median and interquartile range because the treatment intervals showed marked outliers and skewed distributions. Treatment intervals were checked for missing, negative, and implausibly long values. Extreme values were reviewed for plausibility and retained when the underlying dates were internally consistent; no observations were excluded solely on the basis of their magnitude. Age was additionally reported using the mean and range.

Differences in the distribution of initial UICC stages between the three predefined periods were assessed using Pearson’s chi-square test, with Cramér’s V reported as a measure of effect size. Post hoc pairwise comparisons between periods were performed using chi-square tests with Holm correction for multiple testing.

Differences in treatment intervals between the three predefined periods were assessed using the Kruskal–Wallis test. When the global test indicated a statistically significant difference, post hoc pairwise comparisons were performed using the Mann–Whitney U test with Holm correction for multiple testing. Effect sizes for pairwise comparisons were calculated as r = Z/√n and interpreted as weak for r < 0.3, moderate for 0.3 ≤ r ≤ 0.5, and strong for r > 0.5.

As an additional exploratory analysis, initial UICC stage and the three treatment intervals showing significant differences between the predefined periods were evaluated separately according to year of diagnosis from 2018 to 2024. These intervals comprised the time from diagnosis to the first multidisciplinary tumor board, the time from the tumor board to surgery without subsequent radiotherapy, and the time from the tumor board to surgery with subsequent radiotherapy. The annual distribution of UICC stages was assessed using Pearson’s chi-square test, and annual differences in treatment intervals were evaluated using the Kruskal–Wallis test. Pairwise comparisons between adjacent years were performed using the Mann–Whitney U test with Holm correction for multiple testing. All tests were two-sided, and an adjusted p value < 0.05 was considered statistically significant.

## Results

### Study population

Data from 3,216 patients with oral squamous cell carcinoma were obtained from the Cancer Registry of Saxony. According to the date of diagnosis, 1,051 patients (32.7%) were assigned to the pre-pandemic period, 829 patients (25.8%) to the early pandemic period, and 1,336 patients (41.5%) to the later pandemic period.

At diagnosis, patients were aged 28–98 years, with a mean age of 66.14 years. Women accounted for 30.6% of the cohort and had a mean age of 70.77 years. Men accounted for 69.4% of the cohort and had a mean age of 64.10 years. The number of evaluable patients varied between endpoints according to the availability of the required dates or staging information. Baseline demographic and clinical characteristics according to the three predefined periods are presented in **Table 1**.

**Table 1:** Baseline characteristics of the study population according to the predefined pandemic-related periods.

Characteristic	Pre-pandemic before 03/2020	Early pandemic 03/2020-09/2021	Later pandemic after 09/2021	Total
Patients, n	1,051	829	1,336	3,216
Age at diagnosis, years				
Mean +/- SD	65.11 +/- 12.02	65.78 +/- 11.96	67.17 +/- 11.73	66.14 +/- 11.92
Range	30-97	28-94	32-98	28-98
Sex, n (%)				
Female	316 (30.1%)	251 (30.3%)	417 (31.2%)	984 (30.6%)
Male	735 (69.9%)	578 (69.7%)	919 (68.8%)	2,232 (69.4%)
Initial UICC stage, n (%)				
Patients with available UICC stage, n	1,015	803	1,283	3,101
Stage 0	24 (2.4%)	33 (4.1%)	45 (3.5%)	102 (3.3%)
Stage I	245 (24.1%)	199 (24.8%)	331 (25.8%)	775 (25.0%)
Stage II	122 (12.0%)	125 (15.6%)	200 (15.6%)	447 (14.4%)
Stage III	157 (15.5%)	153 (19.1%)	203 (15.8%)	513 (16.5%)
Stage IV	467 (46.0%)	293 (36.5%)	504 (39.3%)	1,264 (40.8%)
Treatment pathways, n (%)				
Patients evaluable across specified pathways, n	709	706	916	2,331
Surgery without subsequent radiotherapy	133 (18.8%)	213 (30.2%)	342 (37.3%)	688 (29.5%)
Radiotherapy without previous surgery	104 (14.7%)	123 (17.4%)	69 (7.5%)	296 (12.7%)
Surgery with subsequent radiotherapy	183 (25.8%)	175 (24.8%)	224 (24.5%)	582 (25.0%)
Systemic therapy	289 (40.8%)	195 (27.6%)	281 (30.7%)	765 (32.8%)

Values are presented as n (%) unless otherwise indicated. Percentages were calculated using the number of patients included in the respective section as the denominator. Treatment categories refer to patients evaluable for the respective treatment pathways. The distribution of initial UICC stages differed significantly between the three predefined periods (Pearson χ²= 26.43, df = 8, p < 0.001; Cramér’s V = 0.065); *post hoc* pairwise comparisons used Pearson’s chi-square tests with Holm correction. SD, standard deviation; UICC, Union for International Cancer Control.

### Treatment intervals according to the predefined periods

The interval from diagnosis to the first multidisciplinary tumor board was available for 2,534 patients (**Figure 1**). The median interval was 17 days [IQR 7–38] in the pre-pandemic period, 15 days [IQR 7–28] in the early pandemic period, and 15 days [IQR 8–27] in the later pandemic period. The global difference between the three periods was statistically significant (Kruskal–Wallis H = 15.71, p < 0.001). After Holm correction, significant differences remained between the pre-pandemic and early pandemic periods (adjusted p < 0.001, r = 0.096) and between the pre-pandemic and later pandemic periods (adjusted p = 0.004, r = 0.071). No significant difference was observed between the early and later pandemic periods (adjusted p = 0.279).

**Figure 1:**
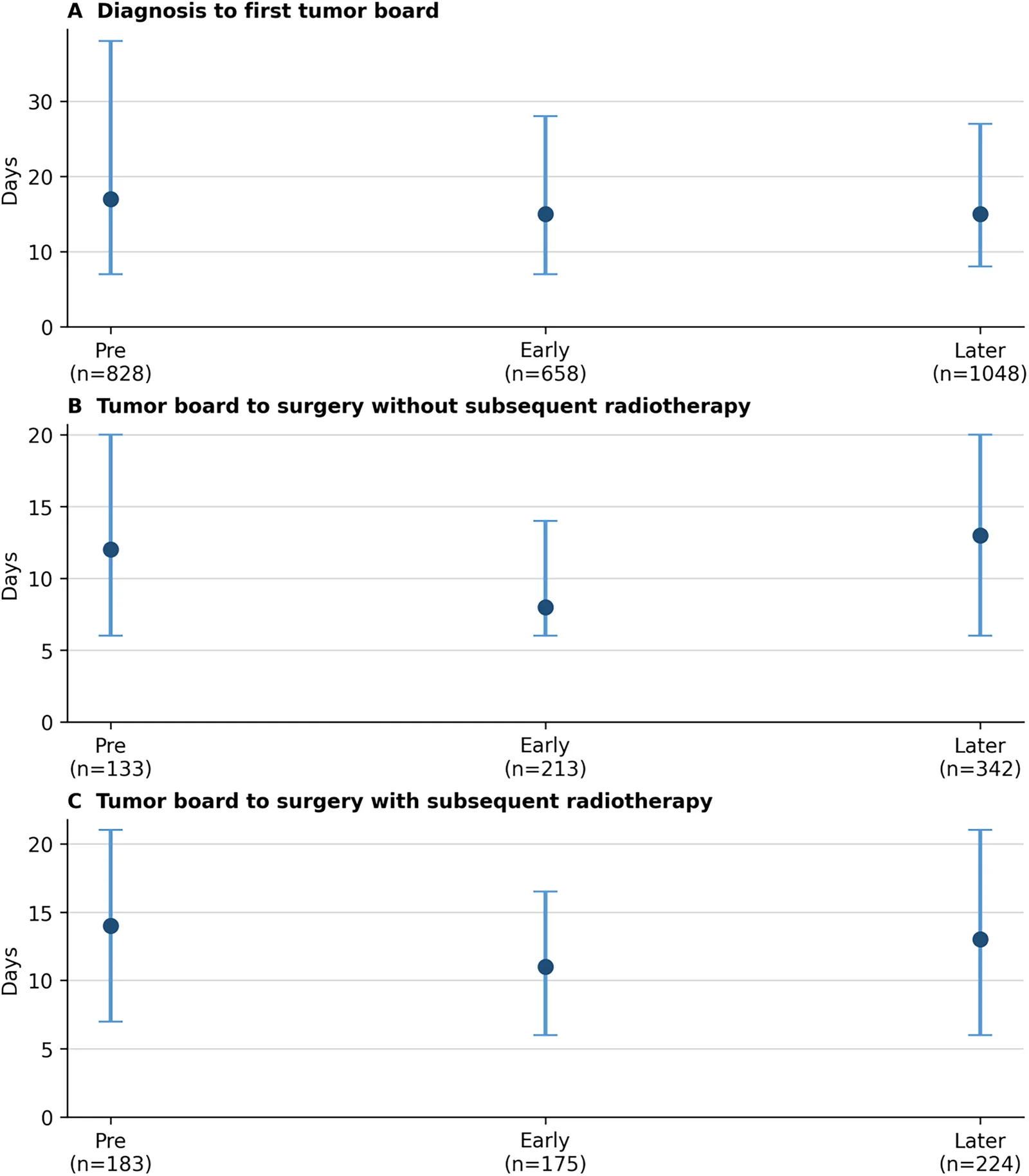
Three vertically arranged plots compare treatment intervals across the pre-pandemic, early pandemic, and later pandemic periods. Panel **(A)** shows diagnosis to first tumor board, with median intervals of approximately 17, 15, and 15 days. Panel **(B)** shows tumor board to surgery without subsequent radiotherapy, with medians of approximately 12, 8, and 13 days. Panel **(C)** shows tumor board to surgery with subsequent radiotherapy, with medians of approximately 14, 11, and 13 days. Points indicate medians; error bars show interquartile ranges. Vertical axes show days, and horizontal axes identify periods and sample sizes.

Among patients undergoing surgery without subsequent radiotherapy, the interval from the tumor board to surgery was available for 688 patients. The median interval was 12 days [IQR 6–20] in the pre-pandemic period, 8 days [IQR 6–14] in the early pandemic period, and 13 days [IQR 6–20] in the later pandemic period. The global difference was statistically significant (Kruskal–Wallis H = 12.27, p = 0.002). After Holm correction, significant differences were observed between the pre-pandemic and early pandemic periods (adjusted p = 0.022, r = 0.137) and between the early and later pandemic periods (adjusted p = 0.002, r = 0.142). The pre-pandemic and later pandemic periods did not differ significantly (adjusted p = 0.971).

Among patients undergoing surgery with subsequent radiotherapy, the interval from the tumor board to surgery was available for 582 patients. The median interval was 14 days [IQR 7–21] in the pre-pandemic period, 11 days [IQR 6–16.5] in the early pandemic period, and 13 days [IQR 6–21] in the later pandemic period. The global difference was statistically significant (Kruskal–Wallis H = 12.24, p = 0.002). After Holm correction, only the comparison between the pre-pandemic and early pandemic periods remained statistically significant (adjusted p = 0.001, r = 0.187). Neither the comparison between the pre-pandemic and later pandemic periods nor that between the early and later pandemic periods was statistically significant (both adjusted p = 0.112).

All statistically significant pairwise differences had weak effect sizes (r < 0.3).

### Initial UICC stage according to the predefined periods

Initial UICC stage was available for 3,101 patients. In the pre-pandemic period, 2.4% of patients had stage 0, 24.1% stage I, 12.0% stage II, 15.5% stage III, and 46.0% stage IV disease. The corresponding proportions were 4.1%, 24.8%, 15.6%, 19.1%, and 36.5% in the early pandemic period and 3.5%, 25.8%, 15.6%, 15.8%, and 39.3% in the later pandemic period.

The distribution of initial UICC stages differed significantly between the three predefined periods (Pearson χ² = 26.43, df = 8, p < 0.001; Cramér’s V = 0.065). In Holm-adjusted pairwise comparisons, significant differences were observed between the pre-pandemic and early pandemic periods (adjusted p < 0.001) and between the pre-pandemic and later pandemic periods (adjusted p = 0.012), whereas no significant difference was observed between the early and later pandemic periods (adjusted p = 0.318). The association between period and UICC stage was weak.

### Year-by-year analysis

As an additional exploratory analysis, outcomes were evaluated according to year of diagnosis. Significant annual variation was observed in the distribution of initial UICC stages (Pearson χ² = 53.88, df = 24, p < 0.001; Cramér’s V = 0.066). The proportion of patients with stage IV disease was 48.1% in 2018, 45.7% in 2019, 36.2% in 2020, 38.7% in 2021, 42.1% in 2022, 35.5% in 2023, and 39.3% in 2024. The annual distribution therefore did not show a uniform monotonic pattern.

The interval from diagnosis to the first multidisciplinary tumor board also differed significantly between years (Kruskal–Wallis H = 27.90, p < 0.001). The annual median decreased from 20 days in 2018 to 15 days in 2020 and 2021, increased to 18 days in 2022, and decreased to 14 days in 2023 and 15 days in 2024.

For surgery without subsequent radiotherapy, the annual median interval from the tumor board to surgery ranged from 8 to 13 days and did not differ significantly between years (Kruskal–Wallis H = 9.37, p = 0.154). For surgery with subsequent radiotherapy, the annual median ranged from 9 to 15 days, with a statistically significant global difference (Kruskal–Wallis H = 13.07, p = 0.042). In the Holm-adjusted comparisons between adjacent years, only the difference between 2019 and 2020 remained statistically significant (adjusted p = 0.008).

Overall, the exploratory year-by-year analysis demonstrated non-linear temporal variation rather than a consistent change at both predefined pandemic-related cut-off points. Detailed annual results are presented in **Supplementary Table S1**. Data for 2024 were available only until 5 August 2024.

## Discussion

A major strength of this study is the large registry-based cohort of 3,216 patients with oral squamous cell carcinoma. Women accounted for 30.6% and men for 69.4% of the cohort, which is consistent with the sex distribution reported in the literature (1, 2). The mean age at diagnosis was 64.10 years for men and 70.77 years for women. The cohort included patients diagnosed during the pre-pandemic, early pandemic, and later pandemic periods, allowing temporal differences in initial UICC stage and post-diagnostic treatment intervals to be evaluated over more than six years.

The principal finding was that the evaluated post-diagnostic treatment intervals were not prolonged during the early pandemic period compared with the pre-pandemic period. Significant differences between the predefined periods were identified for the interval from diagnosis to the first multidisciplinary tumor board and for the intervals from the tumor board to surgery with and without subsequent radiotherapy. However, all corresponding effect sizes were weak, indicating that the magnitude of these differences was limited. Moreover, the median treatment intervals did not show a uniform progressive reduction across the three periods. For example, the interval from the tumor board to surgery without subsequent radiotherapy was shortest in the early pandemic period but increased again in the later pandemic period. These findings do not support a simple interpretation of either pandemic-related deterioration or improvement in post-diagnostic care.

The additional exploratory year-by-year analysis further qualified the comparison of the three predefined periods. The interval from diagnosis to the first multidisciplinary tumor board decreased between 2018 and 2020, remained stable in 2021, increased in 2022, and decreased again in 2023. Similarly, the interval from the tumor board to surgery with subsequent radiotherapy reached its lowest median in 2020 but did not continue to decrease thereafter. No significant annual difference was identified for surgery without subsequent radiotherapy. Thus, the annual patterns were non-linear and did not consistently coincide with both predefined pandemic-related cut-off points. This finding is particularly important because it indicates that differences between the three broad periods may partly reflect secular temporal variation rather than discrete changes attributable to the onset or later course of the pandemic.

Previous studies examining oral and head and neck cancer care during the COVID-19 pandemic have reported heterogeneous findings. In a German cohort of patients with oral cancer, Metzger et al. observed a longer treatment delay and a higher pathological T classification during the first pandemic year compared with the preceding period (17). In contrast, a German multicenter study by Heimes et al. found no clear increase in tumor stage and reported a trend toward a shorter time to intervention during the lockdown period (18). These contrasting observations within the same national healthcare system demonstrate that pandemic-related changes were not necessarily uniform across institutions, regions, or patient populations.

More recent evidence supports this heterogeneous picture. A meta-analysis of studies examining head and neck cancer during the COVID-19 pandemic suggested a modest shift toward more advanced disease at diagnosis, although substantial heterogeneity between the included studies was present (19). Similarly, a systematic review focusing on time to treatment in head and neck cancer identified considerable variation across studies, with prolonged, shortened, or unchanged treatment intervals reported in different healthcare settings (20). The present findings from Saxony therefore add population-based regional evidence to a literature that does not demonstrate a uniform pandemic-related effect on treatment intervals or disease stage.

Several mechanisms may have contributed to the temporal differences observed in the present study. The prioritization of oncological procedures and increasing experience in maintaining healthcare delivery under pandemic conditions may have helped preserve post-diagnostic cancer care despite broader restrictions. The findings of Heimes et al., who observed no clear stage shift and a trend toward shorter time to intervention during the lockdown period, are compatible with the possibility that urgent oncological pathways were maintained in parts of the German healthcare system (18). However, the registry data analyzed in the present study do not permit these mechanisms to be tested directly. Secular developments unrelated to the pandemic, including changes in referral pathways, diagnostic procedures, treatment strategies, organizational processes, and registry documentation, may also have influenced the findings. The shorter intervals observed in selected comparisons should therefore not be interpreted as evidence that the COVID-19 pandemic improved the treatment of OSCC.

The distribution of initial UICC stages also differed significantly between the predefined periods, although the association between period and UICC stage was weak (Cramér’s V = 0.065). Stage IV disease was more frequent in the pre-pandemic period than in the early and later pandemic periods. However, the exploratory year-by-year analysis again showed no uniform trend: the proportion of stage IV disease decreased between 2018 and 2020 but subsequently fluctuated. These findings differ from reports describing a shift toward more advanced disease during the pandemic (17, 19), but are more consistent with studies in which no clear pandemic-related increase in tumor stage was identified (18). The observed differences between studies may reflect variation in study populations, healthcare systems, observation periods, definitions of pandemic phases, tumor sites, and staging procedures.

Importantly, the present findings do not establish that the pandemic led to earlier tumor detection in Saxony. The dataset contains no information on the onset of symptoms, the first contact with a healthcare professional, or the interval between first presentation and histological diagnosis. The study therefore evaluates only the post-diagnostic component of the treatment pathway. This distinction is relevant because delays in oral cancer care may occur at several different stages. Earlier studies documented substantial patient-related and healthcare-related delays before definitive treatment (7–9). A systematic review by Lauritzen et al. further demonstrated considerable heterogeneity in the reported relationships between diagnostic and treatment delays, disease stage, and survival in oral cavity cancer (6).

The absence of longer post-diagnostic treatment intervals during the early pandemic period therefore does not exclude disruption earlier in the diagnostic pathway. During the pandemic, survey data indicated that patients postponed cancer screening, general health examinations, and dental visits (14), while German healthcare data demonstrated substantial reductions in the utilization of several screening and outpatient services (15). Furthermore, clinical cancer registry data from Saxony showed a temporary reduction in newly documented cancer cases during the early pandemic period (16). Such changes could have delayed initial presentation or diagnostic confirmation without necessarily affecting the subsequent treatment intervals captured in the present dataset. Our findings should therefore not be generalized to the entire diagnostic pathway. 

Several limitations must be considered. First, the retrospective observational design does not permit causal attribution of the observed temporal differences to the COVID-19 pandemic. The study identifies associations between calendar periods and clinical parameters but cannot determine whether the pandemic itself caused the observed changes.

Second, allocation of patients to the three predefined periods depends on the selected cut-off points, particularly the pragmatic transition point of September 2021. This cut-off was intended to distinguish the initial phase of acute pandemic-related disruption from a later phase characterized by increasing adaptation of healthcare delivery and was not intended to represent the epidemiological end of the pandemic. Alternative cut-off points might have resulted in different group compositions and estimates. The later pandemic period also included subsequent waves of SARS-CoV-2 infection and should therefore not be interpreted as a post-pandemic period. The additional year-by-year analysis was performed specifically to examine temporal variation independently of these broad predefined periods.

Third, only two complete pre-pandemic years were available, limiting the ability to determine whether temporal trends were already present before March 2020. In addition, data for 2024 were available only until 5 August, resulting in a shorter observation period and potentially incomplete documentation of subsequent treatment steps for patients diagnosed during that year. The number of evaluable patients also differed between endpoints because calculation of each interval required documentation of both relevant dates. Complete-case analyses may therefore be affected by selection bias if the availability of treatment dates differed systematically between periods.

Fourth, the data were obtained from the Cancer Registry of Saxony and included patients treated at different medical centers. This provides a broad regional cohort but may also introduce heterogeneity in documentation and clinical practice. The results may not be directly generalizable to other German states or healthcare systems. Conversely, the registry-based design reduces the dependence on the experience of a single institution and provides complementary evidence to previously published single-center and multicenter studies.

Finally, although Holm correction was applied to post hoc pairwise comparisons, several analyses, particularly the additional year-by-year analyses, were exploratory. Statistical significance should therefore be interpreted together with the magnitude of the observed differences. This is particularly relevant in a large cohort, in which relatively small differences may reach statistical significance. In the present study, the association between period and UICC stage was weak, and all significant pairwise effects for treatment intervals were also weak.

Overall, the present findings do not indicate a deterioration in the evaluated post-diagnostic care of patients with documented OSCC during the early pandemic period. At the same time, they do not provide evidence that the pandemic improved treatment pathways or resulted in earlier tumor detection. The non-linear annual patterns, weak associations, and heterogeneity of findings reported in the wider literature indicate that the observed differences cannot be attributed solely to the COVID-19 pandemic. They are more appropriately interpreted as temporal differences associated with the predefined pandemic-related periods within the context of broader changes in cancer care.

## Conclusion

After categorizing patients according to the predefined pandemic-related periods, no prolongation of the evaluated post-diagnostic treatment intervals was observed during the early pandemic period compared with the pre-pandemic period. The distribution of initial UICC stages differed significantly between the predefined periods, with a higher proportion of stage IV disease in the pre-pandemic period; however, the association between period and UICC stage was weak. These findings should not be interpreted as evidence that the COVID-19 pandemic improved care for patients with OSCC or led to earlier tumor detection.

The additional exploratory year-by-year analysis demonstrated non-linear temporal variation rather than a consistent change at both predefined pandemic-related cut-off points. The observed findings may therefore reflect healthcare adaptation during the pandemic as well as broader secular developments in cancer care. Given the retrospective observational design and the limited observation period in 2024, the findings should be interpreted as temporal associations rather than as causal effects of the COVID-19 pandemic.

## Data Availability

The data analyzed in this study is subject to the following licenses/restrictions: For this retrospective study, we were provided with pseudonymized data from the Cancer Registry of Saxony. Requests to access these datasets should be directed to Michaela Buckova, Michaela.Buckova@ukdd.de.

## References

[1] Robert Koch-Institut Gesellschaft der epidemiologischen Krebsregister in Deutschland e.V . Krebs in Deutschland Für 2019/2020. Berlin: Robert Koch-Institut (2023).

[2] Warnakulasuriya S . Global epidemiology of oral and oropharyngeal cancer. Oral Oncol. (2009) 45:309–16. doi: 10.1016/j.oraloncology.2008.06.002 18804401

[3] Holtmann H Hackenberg B Wilhelm SB Handschel J . Basics Mund-, Kiefer- Und Plastische Gesichtschirurgie. 2. München: Elsevier (2020). Available online at: https://shop.elsevier.de/basics-mund-kiefer-und-plastische-gesichtschirurgie-9783437428470.html (Accessed November 11, 2025).

[4] Jerjes W Upile T Petrie A Riskalla A Hamdoon Z Vourvachis M . Clinicopathological parameters, recurrence, locoregional and distant metastasis in 115 T1-T2 oral squamous cell carcinoma patients. Head Neck Oncol. (2010) 2:9. doi: 10.1186/1758-3284-2-9 20406474 PMC2882907

[5] Pitiphat W Diehl SR Laskaris G Cartsos V Douglass CW Zavras AI . Factors associated with delay in the diagnosis of oral cancer. J Dent Res. (2002) 81:192–7. doi: 10.1177/0810192 11876274

[6] Lauritzen BB Jensen JS Grønhøj C Wessel I von Buchwald C . Impact of delay in diagnosis and treatment-initiation on disease stage and survival in oral cavity cancer: a systematic review. Acta Oncol. (2021) 60:1083–90. doi: 10.1080/0284186x.2021.1931712 34043480

[7] Scully C Malamos D Levers BG Porter SR Prime SS . Sources and patterns of referrals of oral cancer: role of general practitioners. Br Med J (Clinical Res ed). (1986) 293:599–601. doi: 10.1136/bmj.293.6547.599 3092946 PMC1341389

[8] Dimitroulis G Reade P Wiesenfeld D . Referral patterns of patients with oral squamous cell carcinoma, Australia. Eur J Cancer Part B Oral Oncol. (1992) 28:23–7. doi: 10.1016/0964-1955(92)90007-N 1422466

[9] Hollows P McAndrew PG Perini MG . Delays in the referral and treatment of oral squamous cell carcinoma. Br Dent J. (2000) 188:262–5. doi: 10.1038/sj.bdj.4800449 10758688

[10] Jones TM Hargrove O Lancaster J Fenton J Shenoy A Roland NJ . Waiting times during the management of head and neck tumours. J Laryngol Otol. (2002) 116:275–9. doi: 10.1258/0022215021910744 11945188

[11] Böhmer MM Buchholz U Corman VM Hoch M Katz K Marosevic DV . Investigation of a COVID-19 outbreak in Germany resulting from a single travel-associated primary case: a case series. Lancet Infect Dis. (2020) 20:920–8. doi: 10.1016/s1473-3099(20)30314-5 32422201 PMC7228725

[12] World Health Organization . WHO Director-General's opening remarks at the media briefing on COVID-19 – 11 March 2020 Geneva: World Health Organization (2020). Available online at: https://www.who.int/news-room/speeches/item/who-director-general-s-opening-remarks-at-the-media-briefing-on-covid-19---11-march-2020 (Accessed September 10, 2026).

[13] Sächsisches Gesetz- und Verordnungsblatt 37/2020: Verordnung des Sächsischen Staatsministeriums für Soziales und Gesellschaftlichen Zusammenhalt zum Schutz vor dem Coronavirus SARS-CoV-2 und COVID-19 (Sächsische Corona-Schutz-Verordnung – SächsCoronaSchVO). SV SAXONIA Verlag für Recht, Wirtschaft und Kultur GmbH. (2020).

[14] COVID-19 Snapshot Monitoring (COSMO) . Medizinische Versorgung während der COVID-19-Pandemie Universität Erfurt (2020). Available online at: https://projekte.uni-erfurt.de/cosmo2020/web/ (Accessed September 10, 2026).

[15] Mangiapane S Kretschmann J Czihal T von Stillfried D . Veränderung der vertragsärztlichen Leistungsinanspruchnahme während der COVID-Krise: Tabellarischer Trendreport bis zum 1. Halbjahr 2022. In: Veränderung der vertragsärztlichen Leistungsinanspruchnahme. Zentralinstitut für die kassenärztliche Versorgung in Deutschland, Berlin (2022).

[16] Piontek D Klagges S Schubotz B Werner C Wulff J . Documented new cases of cancer in the clinical cancer registries of the german state of saxony during the COVID-19 pandemic. Dtsch Arztebl Int. (2021) 118:328–9. doi: 10.3238/arztebl.m2021.0216 34140084 PMC8295532

[17] Metzger K Mrosek J Zittel S Pilz M Held T Adeberg S . Treatment delay and tumor size in patients with oral cancer during the first year of the COVID-19 pandemic. Head Neck. (2021) 43:3493–7. doi: 10.1002/hed.26858 34494330 PMC8652728

[18] Heimes D Müller LK Schellin A Naujokat H Graetz C Schwendicke F . Consequences of the COVID-19 pandemic and governmental containment policies on the detection and therapy of oral malignant lesions-A retrospective, multicenter cohort study from germany. Cancers (Basel). (2021) 13:2892. doi: 10.3390/cancers13122892 34207863 PMC8227890

[19] Clements K Thapa A Cowell A Conway D Douglas CM Paterson C . Impact of the COVID-19 pandemic on stage and incidence of head and neck cancer: A rapid review and meta-analysis. Clin Otolaryngol. (2024) 49:363–75. doi: 10.1111/coa.14152 38514776

[20] Grumstrup Simonsen M Fenger Carlander AL Kronberg Jakobsen K Grønhøj C Von Buchwald C . The impact of the COVID-19 pandemic on time to treatment in head and neck cancer management: a systematic review. Acta Oncol. (2025) 64:156–66. doi: 10.2340/1651-226x.2025.41366 39876689 PMC11808816

[21] Robert Koch-Institut . Täglicher Lagebericht Des RKI Zur Coronavirus-Krankheit-2019 (COVID-19), 30.09.2021. Berlin: Robert Koch-Institut (2021).

