# Supplementary material for: Temporal variation in initial UICC stage and post-diagnostic treatment intervals in patients with oral squamous cell carcinoma in Saxony before and during the COVID-19 pandemic
Source: Front Oncol. 2026 Oct 19;16:1770029. doi: 10.3389/fonc.2026.1770029 (PMC13724502; doi:10.3389/fonc.2026.1770029)
Supplement: Supplementary file 1 [file DataSheet1.pdf]

Supplementary Table S1. Year-by-year analysis according to year of diagnosis

Parameter	2018	2019	2020	2021	2022	2023	2024	Global p
Patients with available UICC stage	430	481	514	509	473	470	224	<0.001
UICC stage 0, n (%)	8 (1.9%)	13 (2.7%)	20 (3.9%)	22 (4.3%)	15 (3.2%)	21 (4.5%)	3 (1.3%)	
UICC stage I, n (%)	104 (24.2%)	120 (24.9%)	114 (22.2%)	139 (27.3%)	120 (25.4%)	119 (25.3%)	59 (26.3%)	
UICC stage II, n (%)	50 (11.6%)	55 (11.4%)	88 (17.1%)	62 (12.2%)	66 (14.0%)	83 (17.7%)	43 (19.2%)	
UICC stage III, n (%)	61 (14.2%)	73 (15.2%)	106 (20.6%)	89 (17.5%)	73 (15.4%)	80 (17.0%)	31 (13.8%)	
UICC stage IV, n (%)	207 (48.1%)	220 (45.7%)	186 (36.2%)	197 (38.7%)	199 (42.1%)	167 (35.5%)	88 (39.3%)	
Diagnosis to first tumor board, days	20 [8-42] (n=341)	16 [7-35] (n=399)	15 [7-28] (n=425)	15 [6-28] (n=415)	18 [8-31] (n=375)	14 [8-25] (n=383)	15 [8-24.75] (n=196)	<0.001
Tumor board to surgery without subsequent radiotherapy, days	11 [6-18] (n=47)	13 [6-21.75] (n=66)	8 [6-15] (n=125)	8 [6-14] (n=127)	11 [6-20] (n=117)	13 [6-20] (n=128)	13 [6.25-20] (n=78)	0.154
Tumor board to surgery with subsequent radiotherapy, days	14 [7-20] (n=73)	15 [7-21] (n=93)	9 [6-15.25] (n=112)	13 [6.5-20] (n=99)	13 [6-21] (n=94)	11 [6-19] (n=95)	11.5 [6-19] (n=16)	0.042

UICC stages are presented as n (%). Treatment intervals are presented as median [interquartile range], with the number of evaluable patients shown in parentheses. Global p values are based on Pearson's chi-square test for the annual UICC distribution and the Kruskal-Wallis test for treatment intervals. Data for 2024 were available only until 5 August 2024. The effect size for the annual UICC-stage distribution was weak (Cramér's V = 0.066). In Holm-adjusted pairwise comparisons between adjacent years, only the difference between 2019 and 2020 for the interval from the tumor board to surgery with subsequent radiotherapy remained statistically significant (adjusted p = 0.008).

Supplementary Figure S1

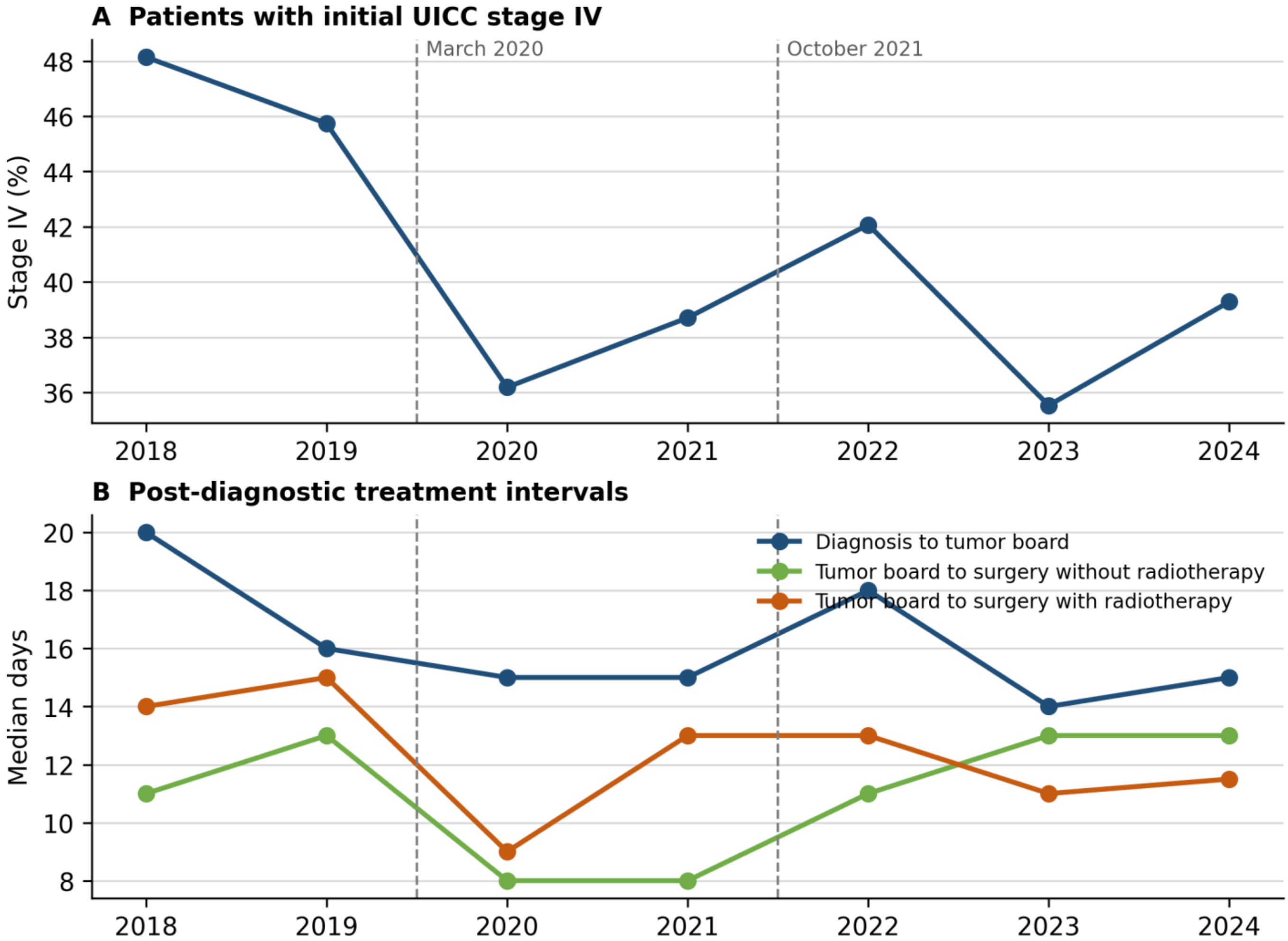

Supplementary Figure S1. Year-by-year variation in initial UICC stage and post-diagnostic treatment intervals

Panel A shows the annual proportion of patients with initial UICC stage IV. Panel B shows annual median treatment intervals. Dashed vertical lines indicate the predefined pandemic-related cut-off points. Data for 2024 were available only until 5 August 2024.
